# Radioprotective Effects of Vitamin C, Cimetidine, and Famotidine on Lipid Peroxidase and Hepatic Glutathione Levels in Mouse Liver

**DOI:** 10.1155/ijcb/1106920

**Published:** 2025-01-04

**Authors:** Mana Gholami, Ali Asghar Ahmadi, Reza Yusofvand, Milad Khanchoupan, Shima Hajimazdarany, Reza Najibi

**Affiliations:** ^1^Department of Biology, Faculty of Sciences, Science and Research Branch, Islamic Azad University, Tehran, Iran; ^2^North Research Center, Pasteur Institute of Iran, Amol, Iran; ^3^Department of Exceptional Talents, Faculty of Medicine Sciences, Lorestan University of Medical Sciences, Khorramabad, Iran; ^4^Department of Chemical Engineering, Faculty of Engineering, University of Urmia, Urmia, Iran; ^5^Department of Biology, Faculty of Science, Babol Branch, Islamic Azad University, Babol, Iran; ^6^Department of Biotechnology and Plant Breeding, Sari Agricultural Sciences and Natural Resources University (SANRU), Sari, Iran

## Abstract

Radiation therapy is one of the most effective treatments for approximately 60% of patients with cancer. During radiation exposure, the overproduction of reactive oxygen species (ROS) disrupts the lipid layer of the membrane, leading to subsequent peroxide radical formation. Cimetidine (Cim) and famotidine (Fam) are histamine H2 receptor antagonists (H2 blocker), also known as peptic ulcer drugs, that exert radioprotective effects. Vitamin C (Vit.C) is an effective free radical and ROS scavenger with significant radioprotective effects. In this experimental study, male mice (6–8 weeks and 28 ± 3 g) were used in five groups. To evaluate ionizing radiation, gamma rays were used at two doses of 2 and 4 Gy and different doses of Cim, Fam, and Vit.C administered as the protectives. Finally, the livers of the mice were isolated and homogenized. The levels of lipid peroxidase and reduced and oxidized glutathione were measured using standard methods. With increasing radiation dose, lipid peroxidase activity, GSSG level, and glutathione content increased. The findings showed that in the drug-only group, Vit.C had better protection than the other two drugs, and the combination of the three drugs had excellent radiation protection. Radiation protection of normal cells in radiotherapy is a valuable necessity. A number of drugs can protect cells against ionizing radiation through different mechanisms. The results suggest that Fam, Cim, and Vit.C can be radioprotective individually or in combination.

## 1. Introduction

Although radiotherapy (RT) is one of the most effective anticancer treatments, ionizing radiation (IR) injuries remain a concern [1]. IR has both immunostimulatory and immunosuppressive effects. The effects of a disease on normal tissues and tumors have many consequences, ranging from mild inflammatory changes to specific forms of programmed cell death. The effect of IR on tissues depends on the physical properties of the IR (type, energy, and the dose rate) [2, 3]. When mammalian cells are exposed to IR, a genetic program is activated, and signaling events involved in DNA repair, cell cycle progression, and apoptosis lead to enhanced survival or cell death [4, 5].

DNA is the primary target of IR, and this radiation reacts with DNA molecules via processes involved in the production of reactive species such as free radicals, and it can also lead to breakage and distortion in DNA strands [6]. Free radicals are highly reactive and unstable chemicals with short half-lives that can be formed from both endogenous and exogenous substances. Free radicals can also initiate a chain of events in the cell, leading to injury and even death [7]. In addition, reactive oxygen species (ROS), such as hydroxyl radicals (OH°: the most destructive), superoxide anion (O_2_) radicals, and other oxidants, such as hydrogen peroxide (H_2_O_2_), are also formed in the presence of oxygen, leading to DNA damage and radiation cytotoxicity in cells [8].

Glutathione is a tripeptide composed of glutamine, cysteine, and glycine, and it plays a critical role in cellular antioxidant defense. It exists in two forms: the reduced form (GSH) and the oxidized form (GSSG). GSH is the active form that participates in various biochemical reactions, primarily as an antioxidant. It donates electrons to neutralize free radicals and ROS, thereby protecting cells from oxidative stress. GSH also plays a vital role in the detoxification of harmful compounds, including heavy metals and xenobiotics, by conjugating with them to facilitate their excretion. In contrast, GSSG is produced when two molecules of GSH undergo oxidation, resulting in the formation of a disulfide bond between the cysteine residues. The ratio of GSH to GSSG within a cell is an important indicator of the cellular redox state and overall oxidative stress. The balance between the reduced and oxidized forms of glutathione is essential for cellular homeostasis, and disturbances in this balance can contribute to various pathological conditions, including cancer, neurodegenerative diseases, and aging. Understanding the dynamics of GSH and GSSG is pivotal for developing therapeutic strategies aimed at enhancing antioxidant defenses and mitigating oxidative damage [9].

Lipid peroxidase (LPO) enzymes, such as lipoxygenases and cyclooxygenases, catalyze the oxidation of polyunsaturated fatty acids, leading to the production of various bioactive lipid mediators, including leukotrienes and prostaglandins. These mediators play crucial roles in inflammation, immune response, and cell signaling. Measuring the levels of malondialdehyde (MDA) or other by-products of lipid peroxidation uses techniques such as the thiobarbituric acid reactive substances (TBARS) assay. Understanding LPO activity is essential for elucidating the mechanisms underlying oxidative stress and its implications for health and disease.

In the mammalian body, there is a balance between endogenous antioxidant defense and free radical/ROS formation, and in an imbalanced condition, it can cause oxidative stress, leading to cell damage [10]. ROS also plays a dual role in promoting ferroptosis through lipid peroxidation while also being regulated by cellular antioxidant mechanisms. Understanding this relationship is crucial for developing targeted therapeutic strategies in diseases where ferroptosis is implicated. Further research is needed to elucidate the precise molecular pathways linking ROS and ferroptosis, which may pave the way for innovative treatments in cancer and other oxidative stress–related diseases.

The production or development of compounds, which are called radioprotector that protect against radiation-induced damage, is one of the major aims of radiobiology. These compounds may be useful in various human exposure situations, such as RT for cancer, radiation workers, people in nuclear accidents, and military personnel in nuclear battles, because a radioprotector maintains the sensitivity of tumor cells to radiation damage and beneficially reduces the radiation effects on healthy normal tissues [11, 12]. Physical shielding is often feasible and not affordable, and according to previous studies on radiation protectors, it is necessary to identify novel, nontoxic, safe, and effective compounds for human protection [13].

Cimetidine (Cim) and famotidine (Fam) are peptic ulcer drugs and histamine H2 receptor antagonists (H2 blocker). However, they also exhibit additional properties that may contribute to their radioprotective effects. Cim has been shown to possess antioxidant properties, which may help reduce oxidative stress induced by radiation. Additionally, it may enhance the immune response and modulate inflammatory pathways, further contributing to its protective effects against radiation-induced damage [14]. Fam is a powerful hydroxyl-radical scavenger that protects against the harmful effects of IR [15, 16]. Ascorbic acid or Vitamin C (Vit.C) is an effective scavenger of free radicals and ROS, and it has significant radioprotective effects. Its ability to donate electrons makes it effective in neutralizing ROS generated by IR. Vit.C also supports the regeneration of other antioxidants, such as Vitamin E, thereby enhancing the overall antioxidant defense system. The radioprotective effects of Vit.C include repairing DNA failure, improving radiation-induced injuries, and inhibiting and reducing apoptosis. However, some reports have indicated that the radioprotective effects of Vit.C are indeterminate; therefore, further research is needed [17].

Preliminary studies suggest that the combined administration of these agents may offer enhanced radioprotection compared to single-agent treatments. For example, studies indicate that the coadministration of Cim and Vit.C results in a more significant reduction in radiation-induced cellular damage, as evidenced by lower levels of oxidative stress markers and improved tissue integrity. Research has demonstrated that both Cim and Fam can provide significant radioprotection when administered alone. Studies have shown that these agents can reduce the incidence of radiation-induced lethality and tissue damage in various animal models. For instance, Cim has been associated with reduced mortality rates and improved survival in mice exposed to lethal doses of radiation. Vit.C has also been shown to mitigate radiation-induced damage, particularly in hematopoietic tissues. Studies indicate that Vit.C administration can enhance the survival of bone marrow cells and improve overall recovery following radiation exposure. The protective effects of Vit.C are attributed to its ability to reduce oxidative stress and promote cellular repair mechanisms.

Furthermore, previous studies reported that radiation exposure after melatonin administration to rats the radiation exposure increased the levels of GSSG and MDA and reduced the level of GSH [18]. The combination of Cim or Fam with Vit.C has been explored to assess potential synergistic effects. The rationale behind this approach lies in the complementary mechanisms of action of these agents; while Cim and Fam may modulate immune responses and reduce inflammation, Vit.C can directly scavenge ROS and enhance antioxidant defenses. Research has also focused on determining the optimal dosing regimens for these agents. The timing of administration (pre- or postradiation exposure) and the specific dosages used can significantly influence the efficacy of radioprotection. Studies have indicated that administering these agents prior to radiation exposure may yield the most pronounced protective effects. The findings regarding the radioprotective effects of Cim, Fam, and Vit.C have important implications for clinical practice, particularly in the context of RT for cancer patients. The use of these agents as adjunctive therapies could potentially enhance the therapeutic index of radiation treatment by protecting normal tissues from damage while allowing for effective tumor control.

In the present study, the radioprotective effects of orally administered Cim, Fam, and Vit.C at single and combined doses on GSH and GSSG levels and LPO (MDA levels) were investigated in the liver of NMRI (Naval Medical Research Institute) mice irradiated with different doses of *γ*-radiation [19].

## 2. Materials and Methods

### 2.1. Animal

In this study, 40 adult male NMRI mice with an age range of 6–8 weeks old and weight range of 28 ± 3 g, purchased from the North Research Center of Pasteur Institute of Iran, Amol, were used (ethical approval Ref. code: NRCIPI-EC/0606168). Animals were housed in polypropylene cages under normal conditions, with the temperature maintained at 21 ± 1°C and humidity maintained at 50%–60%, and the light/dark condition was controlled by automatic lighting (12:12 h, light from 8 to 20). Animals were randomly divided into five groups, and each group was divided into several subgroups. In the control group, no drugs were administered. In the treatment groups, the animals were treated with only one drug (Cim, Fam, or Vit.C), followed by irradiation with 0, 2, or 4 Gy of *γ*-radiation 2 h after the last injection. In the fifth group, the mice were treated with combinations of Cim, Fam, and Vit.C (Fam–Cim, Fam–Vit C, Cim–Vit C, and Fam–Cim–Vit C) and finally irradiated by 0, 2, and 4 Gy of *γ*-radiation 2 h after the last injection [20] (Table 1).

### 2.2. Drug Treatments and Irradiation

Vit.C was provided by Osveh Co. (Tehran, Islamic Republic of Iran), and Fam and Cim were provided by ChemiDaru Co. (Tehran, Islamic Republic of Iran). The mice were individually treated with different concentrations of Fam (0.75, 1.5, and 3 mg/kg), Cim (7.5, 15, and 30 mg/kg), or Vit.C (50, 100, and 200 mg/kg). For the combined use of all three drugs, the optimum protective concentration was selected from the results of the drugs alone because the selected concentrations were closer to the clinical dose.

The optimum concentrations of Fam, Cim, and Vit.C were 1.5, 15, and 100 mg/kg, respectively [21]. For each application, all three drugs were freshly prepared by dissolving in fresh water, and they were administered to mice by gavage. Then, the animals were irradiated with *γ*-rays. Gavage was performed twice a day (12 h) for 3 days before irradiation, and finally, the mice were irradiated with 0, 2, or 4 Gy 2 h after the last dose. The source of the irradiation was cobalt. Each mouse was placed in the irradiation chamber of a gamma cell in a separate clean cage. The animals were irradiated at a dose of rate = 98 cGy/min throughout the irradiation period [22].

Radiation was performed at the Shahid Rajaee Radiotherapy Center, Bipolar, Iran, using 60Co *γ*-rays (Theratron II, 780 C, Canada). The dose rate was 0.98 Gy/min, and the source-to-subject distance (SSD) was 80 cm. Ventilated Plexiglas chambers (12 × 3 × 3 cm) were prepared for each mouse in which they could not move and the whole body was simultaneously exposed to *γ*-rays. The sham group experienced comparable immobilization conditions using the same irradiation chamber. Mice were irradiated for 2–3 h after the last gavage without anesthetization.

### 2.3. Sample Preparation

Forty-eight hours after exposure, mice were sacrificed by cervical vertebral dislocation. Mice in the control group were also killed at the same time period (~48 h after the last gavage). After washing, the mice were in phosphate-buffered saline (PBS) and stored at −80°C for radioprotective study. For homogenization, 1 mL of sucrose buffer + ethylenediaminetetraacetic acid (EDTA) was added to the tubes containing liver tissue, and the samples were homogenized for 3 min using a homogenizer.

### 2.4. The Measurement of LPO by MDA Assay

To homogenize the samples, 1 mL of sucrose buffer + EDTA was added to the tubes containing the liver tissue, and the samples were then homogenized for 3 min using a homogenizer. MDA is a lipid peroxidation end product. MDA levels were determined as previously described by Ohkawa et al. [23] with some modifications. First, 0.2 mL of homogenate was added to 0.2 mL of sodium dodecyl sulfate (SDS) for reaction mixture preparation, and then, 1.5 mL of acetic acid and 1.5 mL of thiobarbituric acid (TBA) were heated at 95°C, for 15 min. An N-butanol-pyridine solution (5 mL) was also added. The mixture was cooled at room temperature, and after centrifugation, the absorbance of the supernatant was measured using a spectrophotometer at a wavelength of 532 nm.

### 2.5. The Measurements of GSH and GSSG

To homogenize the samples, 3 mL of 0.1% EDTA 10 mM + NaClO 450 mM + H_3_PO_4_ buffer was added to the tubes containing liver tissue. The samples were then homogenized for 3 min using a homogenizer. Furthermore, to precipitate the protein, 0.5 mL of a metaphosphoric acid solution was added and centrifuged (5000 *g* for 15 min). Glutathione levels were determined using high-performance liquid chromatography (HPLC) as previously described by Yilmaz et al. [24], with a minor modification. All samples were analyzed by HPLC using a C-18 reversed-phase column, and UV detection was performed at a wavelength of 215 nm and column temperature of 40°C. The aqueous mobile phase was 50 mM NaClO_4_ adjusted to pH 3 with 0.1% H_3_PO_4_. The organic phase was 100% methanol. During the analysis, GSH and GSSG were removed from the chromatographic column during 2.8–3.2 and 4.3–4.8 min, respectively. The results were recorded on a computer, and the chromatogram was plotted.

### 2.6. Statistical Analysis

The data were analyzed using the Statistical Package for the Social Sciences Software Version 19 (Chicago, United States). The results were expressed as mean ± SD, and they were compared using one-way analysis of variance. Differences were considered statistically significant at *p*value < 0.05.

## 3. Results and Discussion

### 3.1. The Effect of Different Doses of Radiation and Drugs on LPO

The optimal protective concentrations of Fam, Cim, and Vit.C were determined in *γ*-irradiated mice. For this purpose, mice were treated with different concentrations of all three drugs and then irradiated with 0, 2, or 4 Gy of radiation. LPO was measured in the liver tissue of mice in different groups.

**Figure 1:**
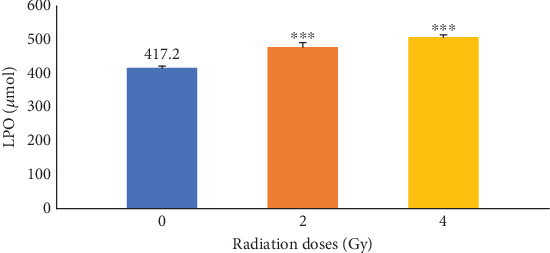
The levels of lipid peroxides activity in control groups receiving only radiation. Increased radiation dose increases lipid peroxides activity. ⁣^∗∗∗^*p* ≤ 0.001 with control groups.

The effects of irradiation on LPO in mice at doses of 0, 2, and 4 were initially determined. The results presented in Figure 1 illustrated that *γ*-irradiation significantly (*p* ≤ 0.001) increases LPO compared with the control group (0 Gy).

As can be seen in Table 2, by increasing the radiation dose, the amount of LPO was significantly increased at different concentrations of Fam (0.75, 1.5, and 3 mg/kg) and Cim (7.5, 15, and 30 mg/kg) alone. However, at a given radiation dose, there was no significant difference in the amount of enzyme activity compared with the control group (0 Gy). Both drugs had significant effect at a *γ*-irradiation dose of 4 Gy and so decreased the lipid peroxidation levels. Exposure to different concentrations of Vit.C (50, 100, and 200 mg/kg) alone demonstrated that enzyme activity increased with increasing radiation dose compared with the control group (0 Gy). There was a significant difference between the group of mice receiving Vit.C (concentrations of 50 and 100 mg/kg) and 2 Gy of radiation and the control group, at similar concentrations. In addition, there was a significant difference between the other group of mice receiving Vit.C (concentrations of 100 and 200 mg/kg) and 4 Gy of radiation and the control group, at similar concentrations.

There was a significant reduction in LPO in the group receiving the combination of Vit.C and Cim with 2 Gy of radiation (VC-2) and the group receiving the combination of Fam and Cim with 2 Gy of radiation (FC-2). The results of the comparison of the three groups showed that there was a significant difference (*p* ≤ 0.001) between the three studied groups, including the group receiving the combination of Vit.C, Cim, and Fam (FVC) with 2 and 4 Gy of radiation, the group receiving the combination of Fam and Cim with 2 and 4 Gy of radiation, and the group receiving the combination of Fam and Vit.C (FV) with 2 and 4 Gy of radiation.

### 3.2. The Effect of Different Doses of Radiation and Drugs on GSH Levels

**Figure 2:**
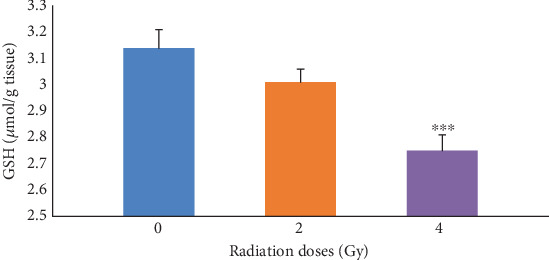
The levels of reduced glutathione (GSH) in control groups receiving only radiation without drugs. Increased radiation dose decreases reduced glutathione (GSH). ⁣^∗∗∗^*p* ≤ 0.001 with control groups.

First, the effects of irradiation at radiation doses of 0, 2, and 4 Gy alone on GSH levels in mice were determined. The results presented in Figure 2 illustrated that, in the groups receiving 4 Gy of radiation, GSH was significantly (*p* ≤ 0.001) decreased compared with the control group (0 Gy). As it can be seen in Table 3, by increasing the radiation dose, the GSH levels decreased in the group receiving Fam and Cim with 4 Gy of radiation compared with the control group (0 Gy). There was a significant difference between the group of mice receiving Vit.C (concentrations of 100 and 200 mg/kg) alone, the group of mice receiving Vit.C (concentrations of 50 mg/kg) with 4 Gy of radiation, and the first control group.

There was a significant difference between the group receiving Vit.C (100 and 200 mg/kg) and 2 Gy of radiation and the control group at similar concentrations. There was also a significant difference between the other group of mice receiving Vit.C (concentrations of 100 mg/kg) and 4 Gy of radiation and the control group, at similar concentrations. In addition, the group receiving the combination of Vit.C and Cim (VC) with 2 and 4 Gy of radiation exhibited a significant difference and increased GSH levels compared with the group receiving the combination of Fam and Cim (FC) with 2 and 4 Gy of radiation.

The results of the comparison of the three groups showed that there was a significant difference (*p* ≤ 0.001) between the three studied groups, including the group receiving the combination of Vit.C and Fam with 2 Gy of radiation, the group receiving the combination of Fam and Cim with 2 and 4 Gy of radiation, and the group receiving the combination of Vit.C and Cim with 2 and 4 Gy of radiation. There was a significant difference between the group receiving the combination of Vit.C, Cim, and Fam with 2 Gy radiation, the group receiving Cim and Fam with 2 Gy radiation, and the group receiving Vit.C and Cim with 2 Gy of radiation.

### 3.3. The Effect of Different Doses of Radiation and Drugs on GSSG Levels

**Figure 3:**
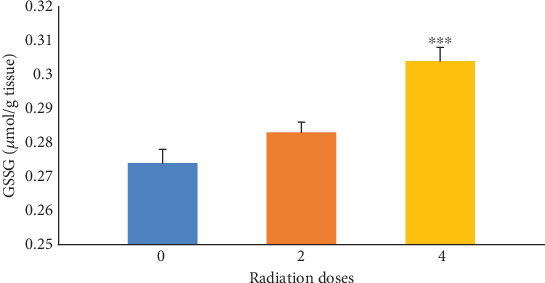
The levels of oxidized glutathione (GSSG) in control groups receiving only radiation without drugs. Increased radiation dose decreases oxidized glutathione (GSSG). ⁣^∗∗∗^*p* ≤ 0.001 with control groups.

Figure 3 indicates the effects of irradiation doses of 0, 2, and 4 Gy alone on GSSG levels in mice. The results illustrated that the GSSG levels were significantly (*p* ≤ 0.001) increased in the group receiving 4 Gy of radiation compared with the control group (0 Gy). As it can be seen in Table 4, by increasing the radiation dose, the amount of GSSG increased in the group receiving Fam or Cim with 4 Gy of radiation compared with the control group (0 Gy).

There were significant differences between the group of mice receiving Vit.C (concentrations of 50, 100, and 200 mg/kg) with 2 and 4 Gy of radiation and the other group of mice receiving Vit.C (concentrations of 100 and 200 mg/kg) alone, in comparison with the first control group. Additionally, there was a significant difference between the group of mice receiving different concentrations of Vit.C (50, 100, and 200 mg/kg) with 2 and 4 Gy irradiation and the control group at similar concentrations. At a dose of 4 Gy radiation, there was a significant difference between all groups receiving the combination of drugs, including the group receiving Vit.C and Cim, the group receiving Vit.C and Fam, and the group receiving Vit.C, Cim, and Fam, compared with the group receiving Cim and Fam at the same radiation dose, and their levels of GSSG were also lower.

The group receiving the combination of Fam and Cim with 4 Gy of radiation was significantly different from the group receiving the combination of Vit.C and Cim and the group receiving the combination of Vit.C and Fam, and the GSSG levels were also increased in this group compared with the other groups. The group receiving Vit.C, Cim, and Fam at 2 Gy had lower GSSG levels, which was significantly different from the group receiving Cim and Fam at 2 Gy and the group receiving Vit.C and Fam at 2 Gy.

## 4. Discussion

Radiation therapy is one of the most effective therapies used for approximately 60% of patients with cancer as part of their treatment regimen. IR, in addition to destroying cancer cells, poisons normal cells, causing cellular damage and unwanted adverse effects [25]. IR affects biological molecules both directly (with a direct impact on DNA) and indirectly (by producing ROS), resulting in cellular damage. During radiation exposure, ROS overproduction disrupts membrane lipids, leading to the formation of peroxide radicals [26]. Oxidative stress is the result of an imbalance between the formation of ROS and the activities of enzymatic and nonenzymatic antioxidants, and it damages all cellular biomolecules [27]. Thus, several defense systems, including nonenzymatic molecules (glutathione, Vitamins A, C, and E, and several antioxidants in foods) as well as enzymatic ROS scavengers, along with superoxide dismutase (SOD), catalase (CAT), and glutathione peroxidase (GPX), have been involved in cells to prevent uncontrolled ROS increase [28]. MDA is a metabolized product of lipid peroxides, and a local increase in its concentration indicates ROS-dependent tissue damage. GPX is an enzyme that reduces H_2_O_2_ in water by converting glutathione from GSH to GSSG. Decreased GSH levels have detrimental consequences on the cellular properties of antioxidant defense [29]. Previous studies have shown that *γ*-rays increase lipid peroxidation activity and decreases hepatic glutathione levels, indicating its harmful effects on the liver [30].

Previous studies have also indicated that histamine H2 receptor antagonists, such as Fam and Cim, are powerful radical scavengers, as well as effective inhibitors of gastric acid secretion. Moreover, Vit.C, an antioxidant, has been confirmed to have a protective effect against internal or external radiation in various organs of mammals. In addition, some reports have indicated that Vit.C's capacity in reducing harmful effects of radiation is inconclusive [31, 32].

In this study, the radioprotective effects of single- and multidrug administration of Cim, Vit.C, and Fam along with *γ*-ray irradiation were evaluated. Moreover, LPO and GSH and GSSG levels were determined to investigate the effects of radiation protection. Our results demonstrated that by increasing the radiation dose, LPO and GSSG levels were significantly increased, whereas GSH levels were significantly decreased, indicating liver damage.

The effects of Fam, Cim, and Vit.C alone and in combination on mice were examined after receiving doses of 0, 2, and 4 Gy of radiation. There was a decrease in LPO in groups of mice receiving Fam (3 mg/kg), Cim (30 mg/kg), and Vit.C (200 mg/kg) singly with 0, 2, and 4 Gy of radiation compared with the drug control (0 concentration of Fam) at the same radiation dose. In the absence of radiation, Cim decreased LPO, which is consistent with the results of Ardestani et al. when they evaluated the effect of 3-day *γ*-irradiation and the protective effects of Cim and ranitidine on mouse serum MDA [33]. In addition, Fam prevents DNA damage by its high scavenging power for HOCl, OH•, and NH_2_Cl [34]. However, in the absence of radiation, there was a significant difference in LPO between all three concentrations of Vit.C compared with similar concentrations in the presence of radiation, and the results showed that the 200 mg/kg concentration was better than the other concentrations. In this regard, the comparison of the radiation protection effects of Cim and Fam using micronuclei in the bone marrow of mice showed that the frequency of micronuclei of polychromatic erythrocyte (MNPCE) was decreased in the treatment groups compared with the control group [21].

There was a significant difference in GSH and GSSG levels between the group of mice receiving Fam (0.75 mg/kg), Cim (15, 30 mg/kg), and Vit.C (50, 100, 200 mg/kg) singly and in the absence of radiation and the group of mice receiving similar concentrations of these three drugs at 4 Gy of radiation. In general, Vit.C increased LPO and GSSG levels and significantly decreased GSH levels. Due to the strong antioxidant properties of Vit.C alone, it was a better radiation protector than the other two drugs.

The comparison of the subgroups of combined group showed the lowest and highest levels of LPO in the combination of Vit.C, Fam, and Cim (FVC) and the combination of Fam and Vit.C (FV) at radiation doses of 2 and 4 Gy, respectively. As a result, the combination of Vit.C, Fam, and Cim at 2 and 4 Gy of radiation had the greatest decreasing effect on LPO compared with the other drug combinations. However, in binary combination of drugs, the combination of Vit.C and Cim (VC) indicated the greatest decrease in LPO compared with the other groups. These results are consistent with those of previous studies [35].

In a study, an investigation of the effects of diphenhydramine (histamine H1 receptor antagonist) and Fam (histamine H2 receptor antagonist) on lipid peroxidation and the activities of antioxidant enzymes showed that diphenhydramine (1 and 5 mM) inhibited the spontaneous lipid peroxidation in the liver and brain of rats, whereas Fam exerted a biphasic concentration-dependent effect (stimulation by 1 mM and inhibition by 5 mM). In addition, an increase and a slight inhibition of SOD enzyme activity were observed in the presence of Fam and diphenhydramine, respectively. However, none of these drugs altered CAT activity [36].

In another study, a survey on the radioprotective effect of genistein in soybean on rats receiving x-rays showed that lipid peroxidation was increased, and liver glutathione levels were decreased in these groups compared with the control group [37]. At 2 Gy of radiation, GSH levels at doses of 0.75 and 1.5 fs and GSSG levels at a dose of 0.75 Fam increased and decreased, respectively, compared with similar concentrations at 4 Gy of radiation. The evaluation of GSSG levels in mice treated with the drug combinations demonstrated that the groups receiving the combination of Vit.C, Fam, and Cim had the lowest levels of GSSG. In the binary combination of drugs, Vit.C and Cim's combination showed the greatest decrease in GSSG levels compared with the other groups.

Additionally, the results regarding the effects of drug combinations on GSH levels indicated that among the mice treated with these combinations, those receiving the combination of Fam, Vit.C, and Cim (FVC) exhibited the lowest levels of GSSG. In the case of binary drug combinations, the combination of Vit.C and Cim (VC) demonstrated the most significant reduction in GSSG levels compared to the other groups. Given that these drugs operate through distinct protective mechanisms, their combination may enhance the body's antioxidant capacity, particularly with respect to glutathione. This mechanism of action is likely attributed to the increased activity of glutathione reductase.

Zivkovic Radojevic et al. concluded the urgent need for strategies to effectively use radioprotectors and mitigators in clinical practice due to the frequent application of IR in medical diagnostics and therapies. Individual patient assessments are recommended to identify the most beneficial compounds for radioprotection [38].

In a study conducted on the oral administration of single- and multidrug assays of Cim, Fam, and Vit.C on micronuclei, it has been indicated that Cim, Fam, and Vit.C demonstrate reliable and similar radioprotective effects, and the single use of these drugs results in responses similar to those of combined forms of drugs [39]. Additionally, in another study, Vitamin A was introduced as a radiation protector. Therefore, we evaluated radiation-induced micronuclei using a micronucleus test. Based on these results, Vitamin A ameliorates the free radicals produced by IR, possibly using the mechanisms used in antioxidants, by reducing genetic damage to tissues and cell destruction in bone marrow [40]. In addition, the protective effect of Poly-MVA (a liquid nanocrystalline compound) on rat livers at 6 Gy of radiation increased liver antioxidant enzyme levels, including glutathione, CAT, and SOD [41].

Research by Guo et al. highlights the multifaceted role of Vit.C in cancer treatments, suggesting its potential to enhance the effectiveness of various therapies, including RT. Lledó et al. had results that Vitamins A, C, D, and E exhibit radioprotective properties, particularly Vitamin E, and call for further research to explore the safety and efficacy of these vitamins against radiation [42, 43]. Jiang et al. demonstrated that Cim offers protective effects against *γ*-rays and neutrons, showing significant improvements in health indicators in rats exposed to combined radiation. This suggests Cim's potential as a radioprotector for patients undergoing RT [44]. Rahgoshai et al. evaluated the radioprotective effects of Cim and IMOD on human lymphocyte cells, finding that both significantly reduced cellular damage from radiation, with hybrid radioprotectors showing the highest efficacy [45]. Naeeji et al. studied the radioprotective effects of Vit.C, Cim, and Fam in mice, concluding that these compounds provided comparable protection, whether used alone or in combination [46]. Lastly, Dizaj assessed the combined effects of Fam and Cim on irradiated mice, finding that while the combination showed a higher dose reduction factor for radioprotection, it did not significantly outperform the individual drugs, indicating no synergistic effect [16].

## 5. Conclusions

In summary, orally administered Cim, Fam, and Vit.C demonstrate promising radioprotective effects, both individually and in combination. Their mechanisms of action involve antioxidant properties, modulation of immune responses, and reduction of oxidative stress. Further research is warranted to elucidate optimal dosing strategies and to explore the potential clinical applications of these agents in protecting against radiation-induced damage. As our understanding of these compounds evolves, they may play a crucial role in improving patient outcomes in radiation therapy and other contexts involving radiation exposure. The results of the oral administration of Fam, Cim, and Vit.C as radioprotective agents prior to irradiation indicate that Vit.C yielded superior results compared to the others. In groups receiving the combination of all three drugs, this combination exhibited enhanced effects in reducing LPO and GSSG levels, while increasing GSH levels. These findings underscore the importance of further research into the efficacy and safety of these radioprotective agents in clinical applications.

## Figures and Tables

**Table 1:** Study design and 38 groups involved in this study.

**Drugs**	**Doses (mg/kg BW)**	**Gamma radiation**		
		**Not radiated**	**2 Gy**	**4 Gy**
Control	0	0–0^a^	0–2	0–4

Fam	0/75	F 0/75–0^a^	F 0/75–2	F 0/75–4
	1/5	F 1/5–0	F 1/5–2	F 1/5–4
	3	F 3–0	F 3–0	F 3–0

Cim	7/5	C 7/5–0	C 7/5–2	C 7/5–4
	15	C 15–0	C 15–2	C 15–4
	30	C 30–0	C 30–2	C 30–4

Vit.C	50	V 50–0	V 50–2	V 50–4
	100	V 100–0	V 100–2	V 100–4
	200	V 200–0	V 200–2	V 200–4

Combined^b^	Fam 1/5 + Cim 15		FC-2	FC-4
	Vit.C 100 + Cim 15		VC-2	VC-4
	Fam 1/5 + Vit.C 100		FV-2	FV-4
	Fam 1/5 + Vit.C 100 + Cim 15		FVC-2	FVC-4

^a^All samples encoded. Each group had an appropriate code.

^b^The groups treated by combination of two or all three drugs, designed and treated after the first phase of study, when we studied single drugs.

**Table 2:** Lipid peroxidase (LPO) activity (micromole) of mice treated with Fam, Cim, and Vit.C as a single dose and in combination with each other, irradiated by *γ*-ray at 0, 2, and 4 Gy.

**Drugs**	**Doses (mg/kg BW)**	**Gamma radiation**		
		**Not radiated**	**2 Gy**	**4 Gy**
Control	0	417/3 ± 10/81	479/2^c^ ±9/70	509/0^c^ ±16/38

Fam	0/75	422/1 ± 8/10	470/4^c^ ±18/03	499/7^c^ ±11/78
	1/5	418/1 ±16/46	459/0^a^±28/27	491/8^c^ ±22/05
	3	410/3 ± 19/01	475/7^c^ ±9/56	507/7^c^ ±20/43

Cim	7/5	402/0 ± 7/16	471/9 ± 7/97	511/0^c^ ±27/02
	15	407/1 ±22/29	480/3^c^ ±9/98	498/6^c^ ±37/78
	30	415/8 ±4/23	449/9^a^±21/29	500/8^c^ ±27/72

Vit.C	50	391/1 ± 8/75	441/1^d^ ±33/72	502/3^cd^ ±23/95
	100	383/1 ± 12/10	436/1^e^ ±23/47	475/4^cf^ ±39/20
	200	367/9^b^ ±4/78	443/0 ±9/12	464/6^b^ ±24/91

Combined	FC		465/6^d^ ±21/28	426/7 ± 31/94
	VC		483/3^ah^ ±13/88	412/7^g^ ±29/12
	FV		487/1^e^ ±11/43	450/8^d^ ±22/87
	FVC		493/6^bi^ ±41/23	441/1^ah^ ±18/19

^a^ *p* ≤ 0.05 with the control group.

^b^ *p* ≤ 0.01 with the control group.

^c^ *p* ≤ 0.001 with the control group.

^d^ *p* ≤ 0.05 with control at similar dose.

^e^ *p* ≤ 0.01 with control at similar dose.

^f^ *p* ≤ 0.001 with control at similar dose.

^g^ *p* ≤ 0.05 with FV at similar dose.

^h^ *p* ≤ 0.01 with FV at similar dose.

^i^ *p* ≤ 0.001 with FV at similar dose.

**Table 3:** Reduced glutathione (GSH) levels (micromole/gram liver tissue) of mice treated with Fam, Cim, and Vit.C as a single dose and in combination with each other, irradiated by *γ*-ray at 0, 2, and 4 Gy.

**Drugs**	**Doses (mg/kg BW)**	**Gamma radiation**		
		**Not radiated**	**2 Gy**	**4 Gy**
Control	0	3/147 ±0/183	3/013 ±0/017	2/753^c^ ±0/511

Fam	0/75	3/013 ±0/149	2/987 ±0/160	2/682^c^ ±0/376
	1/5	3/041 ±0/077	3/102 ±0/304	2/783^b^ ±0/561
	3	2/965 ±0/126	2/915 ±0/314	2/853^a^±0/236

Cim	7/5	3/096 ±0/228	2/997 ±0/317	2/858^a^±0/088
	15	3/183 ±0/159	3/017 ±0/109	2/792^a^±0/138
	30	3/143 ±0/250	3/050 ±0/362	2/845^a^±0/309

Vit.C	50	3/247 ±0/102	3/180 ±0/208	2/818^c^ ±0/327
	100	3/454^b^ ±0/072	3/271^d^ ±0/353	3/014^e^ ±0/373
	200	3/536^c^ ±0/246	3/298^d^ ±0/325	2/974 ±0/297

Combined	FC		3/011^f^ ±0/254	3/321 ±0/090
	VC		3/244^c^ ±0/297	3/297^a^±0/329
	FV		2/867^af^ ±0/211	3/143^a^±0/362
	FVC		3/097^bf^ ±0/115	3/075 ±0/361

^a^ *p* ≤ 0.05 with the control group.

^b^ *p* ≤ 0.01 with the control group.

^c^ *p* ≤ 0.001 with the control group.

^d^ *p* ≤ 0.05 with control at similar dose.

^e^ *p* ≤ 0.01 with control at similar dose.

^f^ *p* ≤ 0.001 with control at similar dose.

**Table 4:** Oxidized glutathione (GSSG) levels (micromole/gram liver tissue) of mice treated with Fam, Cim, and Vit.C as a single dose and in combination with each other, irradiated by *γ*-ray at 0, 2, and 4 Gy.

**Drugs**	**Doses**	**Gamma radiation**		
		**Not radiated**	**2 Gy**	**4 Gy**
	0	0/274 ±0/030	0/284 ±0/009	0/304^c^ ±0/054

Fam	0/75	0/270 ±0/017	0/271 ±0/019	0/307^c^ ±0/056
	1/5	0/270 ±0/009	0/278 ±0/018	0/293 ±0/022
	3	0/277 ±0/025	0/282 ±0/015	0/288 ±0/031

Cim	7/5	0/275 ±0/019	0/269 ±0/022	0/294 ±0/010
	15	0/264 ±0/015	0/271 ±0/011	0/287 ±0/023
	30	0/280 ±0/009	0/282 ±0/016	0/296^b^ ±0/024

Vit.C	50	0/262 ±0/015	0/240^cf^ ±0/027	0/254^af^ ±0/003
	100	0/242^b^ ±0/022	0/248^bf^ ±0/032	0/242^cf^ ±0/002
	200	0/229^c^ ±0/025	0/243^bf^ ±0/027	0/239^cf^ ±0/038

Combined	FC		0/273 ±0/041	0/273 ±0/059
	VC		0/278 ±0/045	0/236^b^ ±0/038
	FV		0/300 ±0/064	0/256^a^±0/009
	FVC		0/263^ah^ ±0/011	0/246^cg^ ±0/042

^a^ *p* ≤ 0.05 with the control group.

^b^ *p* ≤ 0.01 with the control group.

^c^ *p* ≤ 0.001 with the control group.

^f^ *p* ≤ 0.001 with control at similar dose.

^g^ *p* ≤ 0.05 with FV at similar dose.

^h^ *p* ≤ 0.01 with FV at similar dose.

## Data Availability

The authors provide only a subset of data in the paper for space concerns; all other data are available upon request.

## References

[1] Mun G.-I., Kim S., Choi E., Kim C. S., Lee Y.-S. (2018). Pharmacology of natural radioprotectors. *Archives of Pharmacal Research*.

[2] Demaria S., Golden E. B., Formenti S. C. (2015). Role of local radiation therapy in cancer immunotherapy. *JAMA Oncology*.

[3] Liu W., Chen Q., Wu S. (2015). Radioprotector WR-2721 and mitigating peptidoglycan synergistically promote mouse survival through the amelioration of intestinal and bone marrow damage. *Journal of Radiation Research*.

[4] Kratochwil C., Giesel F. L., Heussel C.-P. (2020). Patients resistant against PSMA-targeting *α*-radiation therapy often harbor mutations in DNA damage-repair–associated genes. *Journal of Nuclear Medicine*.

[5] Raee P., Tan S. C., Najafi S. (2023). Autophagy, a critical element in the aging male reproductive disorders and prostate cancer: a therapeutic point of view. *Reproductive Biology and Endocrinology*.

[6] Chatzipapas K. P., Papadimitroulas P., Emfietzoglou D. (2020). Ionizing radiation and complex DNA damage: quantifying the radiobiological damage using Monte Carlo simulations. *Cancers*.

[7] Adjemian S., Oltean T., Martens S. (2020). Ionizing radiation results in a mixture of cellular outcomes including mitotic catastrophe, senescence, methuosis, and iron-dependent cell death. *Cell Death & Disease*.

[8] Pansare K., Vaid A., Singh S. R. (2022). Effect of cold atmospheric plasma jet and gamma radiation treatments on gingivobuccal squamous cell carcinoma and breast adenocarcinoma cells. *Plasma Chemistry and Plasma Processing*.

[9] Ziapour P., Ataee R., Shadifar M. (2011). New intracellular and molecular aspects in pathophysiology of colorectal cancer. *Gastroenterology and Hepatology from Bed to Bench*.

[10] Weitzel D. H., Tovmasyan A., Ashcraft K. A. (2015). Radioprotection of the brain white matter by Mn (III) N-butoxyethylpyridylporphyrin–based superoxide dismutase mimic MnTnBuOE-2-PyP5+. *Molecular Cancer Therapeutics*.

[11] Ping X., Zhang W.-B., Cai X.-H. (2014). Flavonoids of Rosa roxburghii Tratt act as radioprotectors. *Asian Pacific Journal of Cancer Prevention*.

[12] Khafaji M., Kouzaba K., Tashkandi A., Almusallam S., Ghoneim A., Hagi S. (2022). Radiation protection: Are pediatrics health care providers Up to date?. *Medical Science*.

[13] Shahbazi-Gahrouei D., Baradaran-Ghahfarokhi M. (2012). Investigation of patient dose from common radiology examinations in Isfahan, Iran. *Advanced Biomedical Research*.

[14] Antonela Díaz Nebreda (2019). Involvement of histamine H1 and H2 receptor inverse agonists in receptor’s crossregulation. *European Journal of Pharmacology*.

[15] Shahidi M., Mozdarani H. (2003). Potent radioprotective effect of therapeutic doses of ranitidine and famotidine against gamma-rays induced micronuclei in vivo. *International Journal of Radiation Research*.

[16] Dizaj K. A. (2021). Combined effect of oral famotidine and cimetidine on the survival of lethally irradiated mice. *Journal of Cancer Research and Therapeutics*.

[17] Ostróżka-Cieślik A. (2022). The effect of antioxidant added to preservation solution on the protection of kidneys before transplantation. *International Journal of Molecular Sciences*.

[18] Najafi M., Cheki M., Hassanzadeh G., Amini P., Shabeeb D., Musa A. E. (2019). The radioprotective effect of combination of melatonin and metformin on rat duodenum damage induced by ionizing radiation: a histological study. *Advanced Biomedical Research*.

[19] Gholami M., Ahmadi A. A., Hajimazdarany S., Najibi R., Amol I., Gholami F. (2023). Radioprotective effects of vitamin C, cimetidine, and famotidine on lipid peroxidase and hepatic glutathione levels in mouse liver. *1st International Congress on New Approaches of Life Style, Prevention and Treatment of Cancer*.

[20] Fatehi D., Mohammadi M., Shekarchi B., Shabani A., Seify M., Rostamzadeh A. (2018). Radioprotective effects of Silymarin on the sperm parameters of NMRI mice irradiated with *γ*-rays. *Journal of Photochemistry and Photobiology B: Biology*.

[21] Ebrahimi M., Mozdarani H. (2001). A comparative study on the radioprotective effect of cimetidine and famotidine against gamma rays in bone marrow cells. *Physiology and Pharmacology*.

[22] El-Desouky W. (2017). Radioprotective effect of green tea and grape seed extracts mixture on gamma irradiation induced immune suppression in male albino rats. *International Journal of Radiation Biology*.

[23] Ohkawa H., Ohishi N., Yagi K. (1978). Reaction of linoleic acid hydroperoxide with thiobarbituric acid. *Journal of Lipid Research*.

[24] Yilmaz Ö., Keser S., Tuzcu M. (2009). A practical HPLC method to measure reduced (GSH) and oxidized (GSSG) glutathione concentrations in animal tissues. *Journal of Animal and Veterinary Advances*.

[25] Moding E. J., Kastan M. B., Kirsch D. G. (2013). Strategies for optimizing the response of cancer and normal tissues to radiation. *Nature Reviews Drug Discovery*.

[26] Lam P.-L., Rs-M Wong K.-H., Lam L.-K. H. (2020). The role of reactive oxygen species in the biological activity of antimicrobial agents: an updated mini review. *Chemico-Biological Interactions*.

[27] Zhao X., Wang L.-Y., Li J.-M. (2021). Redox‐mediated artificial non-enzymatic antioxidant MXene nanoplatforms for acute kidney injury alleviation. *Science*.

[28] Marrocco I., Altieri F., Peluso I. (2017). Measurement and clinical significance of biomarkers of oxidative stress in humans. *Oxidative Medicine and Cellular Longevity*.

[29] Kwiecien S., Jasnos K., Magierowski M. (2014). Lipid peroxidation, reactive oxygen species and antioxidative factors in the pathogenesis of gastric mucosal lesions and mechanism of protection against oxidative stress-induced gastric injury. *Journal of Physiology and Pharmacology*.

[30] Alkhalf M. I., Khalifa F. K. (2018). Blueberry extract attenuates *γ*-radiation-induced hepatocyte damage by modulating oxidative stress and suppressing NF-*κ*B in male rats. *Saudi Journal of Biological Sciences*.

[31] Monajjemzadeh F., Robertson T. A. (2022). Influencing factors in N-nitrosodimethylamine (NDMA) impurity detection in ranitidine and possible reactivity of other histamine H 2 receptor antagonists. *Journal of Pharmaceutical Innovation*.

[32] Khawaja M., Thakker J., Kherallah R. (2024). Antacid therapy in coronary artery disease and heart failure: proton pump inhibitors vs. H_2_ receptor blockers. *Cardiovascular Drugs and Therapy*.

[33] Ardestani S. K., Janlow M. M., Tavakoli A. K. Z. (2004). Effect of cimetidine and ranitidine on lipid profile and lipid peroxidation in *γ*-irradiated mice. *Acta Medica Iranica*.

[34] Qian Y., Huang J., Liu X. (2020). Rapid oxidation of histamine H2-receptor antagonists by peroxymonosulfate during water treatment: kinetics, products, and toxicity evaluation. *Water Research*.

[35] Alshihri A. (2020). *The Protective Effects of Natural Antioxidants on Ionizing Radiation-Induced Apoptosis and Cell Cycle Arrest in Human Intestinal Epithelial Cells*.

[36] Evazalipour M., Moayedi S., Kozani P. S., Kozani P. S. (2021). The protective effects of carvacrol on diphenhydramine-induced genotoxicity in human peripheral blood lymphocytes. *Research Journal of Pharmacognosy*.

[37] Uslu G. H., Canyilmaz E., Serdar L., Ersöz Ş. (2019). Protective effects of genistein and melatonin on mouse liver injury induced by whole-body ionising radiation. *Molecular and Clinical Oncology*.

[38] Radojevic M. Z. (2023). Review of compounds that exhibit radioprotective and/or mitigatory effects after application of diagnostic or therapeutic ionizing radiation. *International Journal of Radiation Biology*.

[39] Zangeneh M., Mozdarani H., Mahmoudzadeh A. (2015). Potent radioprotective effects of combined regimens of famotidine and vitamin C against radiation-induced micronuclei in mouse bone marrow erythrocytes. *Radiation and Environmental Biophysics*.

[40] Changizi V., Haeri S. A., Abbasi S., Rajabi Z., Mirdoraghi M. (2019). Radioprotective effects of vitamin A against gamma radiation in mouse bone marrow cells. *MethodsX*.

[41] El-Marakby S. M., Selim N. S., Desouky O. S., Ashry H. A., Sallam A. M. (2013). Effects of Poly-MVA on the rheological properties of blood after in-vivo exposure to gamma radiation. *Journal of Radiation Research and Applied Sciences*.

[42] Guo D., Liao Y., Na J. (2024). The involvement of ascorbic acid in cancer treatment. *Molecules*.

[43] Lledó I., Ibáñez B., Melero A. (2023). Vitamins and radioprotective effect: a review. *Antioxidants*.

[44] Jiang D. W., Wang Q. R., Shen X. R. (2017). Radioprotective effects of cimetidine on rats irradiated by long-term, low-dose-rate neutrons and ^60^Co *γ*-rays. *Military Medical Research*.

[45] Rahgoshai S., Mehnati P., Aghamiri M. R. (2021). Evaluating the radioprotective effect of cimetidine, IMOD, and hybrid radioprotectors agents: an in-vitro study. *Applied Radiation and Isotopes*.

[46] Naeeji A., Mozdarani H., Shabestani Monfared A. (2017). Oral administration of vitamin C, cimetidine and famotidine on micronuclei induced by low dose radiation in mouse bone marrow cells. *Journal of Biomedical Physics & Engineering*.

